# Supplementary material for: The microbiome biomarkers of pregnant women’s vaginal area predict preterm prelabor rupture in Western China
Source: Front Cell Infect Microbiol. 2024 Oct 31;14:1471027. doi: 10.3389/fcimb.2024.1471027 (PMC11560878; doi:10.3389/fcimb.2024.1471027)
Supplement: Supplementary file 1 [file DataSheet1.zip › compare_1/Community/Pieplot/order/sample/P18.order.pieplot.pdf]

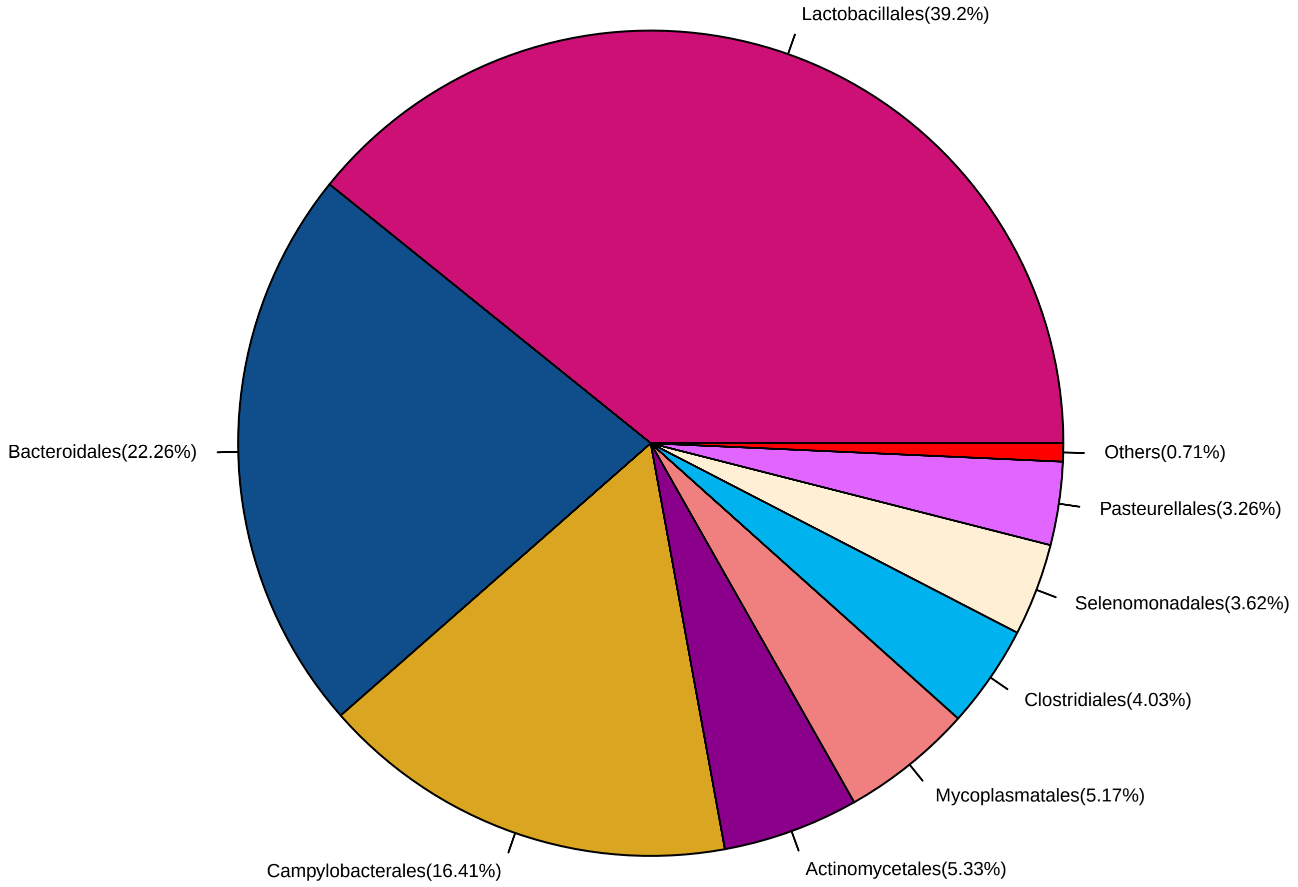
Lactobacillales(39.2%)
Bacteroidales(22.26%)
Others(0.71%)
Pasteurellales(3.26%)
Selenomonadales(3.62%)
Clostridiales(4.03%)
Mycoplasmatales(5.17%)
Actinomycetales(5.33%)
Campylobacterales(16.41%)
